# Therapeutic Effect of Intravenous Infusion of Perfluorocarbon Emulsion on LPS-Induced Acute Lung Injury in Rats

**DOI:** 10.1371/journal.pone.0087826

**Published:** 2014-01-28

**Authors:** Shike Hou, Hui Ding, Qi Lv, Xiaofeng Yin, Jianqi Song, Ning Xu Landén, Haojun Fan

**Affiliations:** 1 Rescue Medical Research Institute, Affiliated Hospital of Logistics University of Chinese People's Armed Police Forces, Tianjin, P.R. China; 2 Molecular Dermatology Research Group, Center for Molecular Medicine (CMM), Karolinska University Hospital, Stockholm, Sweden; The Ohio State University, United States of America

## Abstract

Acute lung injury (ALI) and its more severe form, acute respiratory distress syndrome (ARDS) are the leading causes of death in critical care. Despite extensive efforts in research and clinical medicine, mortality remains high in these diseases. Perfluorocarbon (PFC), a chemical compound known as liquid ventilation medium, is capable of dissolving large amounts of physiologically important gases (mainly oxygen and carbon dioxide). In this study we aimed to investigate the effect of intravenous infusion of PFC emulsion on lipopolysaccharide (LPS) induced ALI in rats and elucidate its mechanism of action. Forty two Wistar rats were randomly divided into three groups: 6 rats were treated with saline solution by intratracheal instillation (control group), 18 rats were treated with LPS by intratracheal instillation (LPS group) and the other 18 rats received PFC through femoral vein prior to LPS instillation (LPS+PFC group). The rats in the control group were sacrificed 6 hours later after saline instillation. At 2, 4 and 6 hours of exposure to LPS, 6 rats in the LPS group and 6 rats in LPS+PFC group were sacrificed at each time point. By analyzing pulmonary pathology, partial pressure of oxygen in the blood (PaO2) and lung wet-dry weight ratio (W/D) of each rat, we found that intravenous infusion of PFC significantly alleviated acute lung injury induced by LPS. Moreover, we showed that the expression of pulmonary myeloperoxidase (MPO), intercellular adhesion molecule-1 (ICAM-1) of endothelial cells and CD11b of polymorphonuclear neutrophils (PMN) induced by LPS were significantly decreased by PFC treatment *in vivo*. Our results indicate that intravenous infusion of PFC inhibits the infiltration of PMNs into lung tissue, which has been shown as the core pathogenesis of ALI/ARDS. Thus, our study provides a theoretical foundation for using intravenous infusion of PFC to prevent and treat ALI/ARDS in clinical practice.

## Introduction

Acute lung injury/acute respiratory distress syndrome (ALI/ARDS), which were firstly described in 1967, clinically manifest as respiratory distress and refractory hypoxemia [1]. The condition is defined by a series of pathological changes, including pneumonedema and micro-atelectasis, which are caused by diffuse injury of alveolar-capillary membrane due to severe infection, trauma, shock, acidosis or toxic inhalation [2], [3]. As a clinical complication of severe ALI, ARDS is a leading cause of morbidity and mortality in critically ill patients [4], [5]. It is initiated by injury to the lung, often in the setting of pneumonia or sepsis, which can result in pulmonary edema and significant hypoxemia [3]. Although positive end-expiratory pressure (PEEP) [6], high frequency oscillatory ventilation (HFOV) [7], [8] and prone positioning [9], [10] have been used to relieve the severe hypoxemia due to ALI/ARDS, none of these interventions is able to decrease mortality in randomized clinical trials [3], [7]–[9]. Despite extensive efforts have been made in experimental and clinical medicine, there are no effective pharmacological treatments for ALI/ARDS existing to date. Multiple therapeutic attempts, including inhaled nitric oxide [11], [12], corticosteroids [13], β2-agonists [14], [15], surfactants [16], and immunomodulating agents such as IL-10 [17] have failed.

Perfluorocarbon (PFC), a molecule consisting wholly of fluorine and carbon, is characterized by high gas solubility, fast release, low surface tension, high volume-quality, average volatility, good histocompatibility and the absence of absorption and metabolism *in vivo* [18], [19]. Because its capability of dissolving large amounts of physiologically important gases (mainly oxygen and carbon dioxide), PFC has been used for liquid breathing medium [20], [21]. Liquid breathing has been proposed as a means of improving gas exchange in infants with acute respiratory failure since the 1970s. Greenspan *et al.* reported that application of liquid perfluorochemical ventilation in human preterm neonates with ARDS resulted in marked improvement in lung distensibility and oxygenation [22]. Partial liquid ventilation (PLV) using PFC and vaporized PFC inhalation have also been proven to improve gas exchange and survival in infants with severe respiratory distress syndrome [23]–[25]. However, these interventions need establishment of artificial airway to facilitate PFC into the lungs, which is not suitable for treating patients with early ALI.

PFC emulsions with exceptionally small particles, which could be infused intravenously, have been clinically evaluated as artificial oxygen carriers to reduce allogeneic blood transfusions or to improve tissue oxygenation [26]. Fluosol DA-20%, a 20% w/v PFC emulsion comprising 14% w/v perfluorodecalin and 6% w/v perfluorotripropylamine emulsified primarily with a synthetic poloxamer, was the first successful commercial development of an injectable PFC emulsion. It was approved for intravenous use by the United States Food and Drug Administration as “oxygen therapeutic” for treatment of myocardial ischemia at the time of balloon angioplasty in 1989 [27]. However, it was withdrawn in 1992 because of cumbersome preparation (stored frozen) and the application of autoperfusion catheters, which make it no need to use PFC during balloon angioplasty [28], [29]. However, due to the multiple potential use of PFC, on-going efforts continue to try to get a safe PFC for use in humans. Recently, Oxygent, an improved second-generation concentrated PFC emulsion based on perflubron (perfluorooctyl bromide; C8F17Br), was developed, which has a shelf-life of up to 2 years at 2°C to 8°C. Oxygent is initially designed for use as a temporary intravenous oxygen carrier, but there are many attractive potential use for Oxygent, such as to treat tissue ischemia, augment tumor PO_2_ levels to enhance sensitivity to radiation and chemotherapy, preserve tissues and prolong storage time of an organ (e.g., kidney) prior to transplantation [29]. Previous studies have shown that PFC can be incorporated in breathing medium during liquid ventilation in patients or experimental animals with ALI/ARDS [23]–[25]. However, it is not known whether intravenous infusion of PFC emulsions can protect from ALI when administered at the early stage of disease process.

In this study, we investigated the effect of intravenous infusion of PFC emulsion on LPS induced ALI in rats and explored the potential molecular mechanisms of its action. Our results demonstrated that PFC infusion could significantly relieve LPS induced acute lung injury, improve gas exchange, and attenuate inflammatory reactions.

## Materials and Methods

### Ethics statement

All animals received humane care in compliance with the “Guide for the Care and Use of Laboratory Animals” published by the National Institutes of Health. The study protocol was approved by the Laboratory Animal Ethics Committee of Affiliated Hospital of Logistical College of Chinese People's Armed Police Forces. All surgery was performed under sodium pentobarbital anesthesia, and all efforts were made to minimize suffering.

### PFC emulsion

PFC emulsion is purchased from Double Crane Pharmacetuicals, Beijing, China, which is an improved second-generation concentrated PFC emulsion based on perflubron (perfluorooctyl bromide; C8F17Br). The current 60% w/v perflubron-based formulation (AF0144) is emulsified with phospholipid as the only surfactant, has an initial median particle diameter of 0.16 to 0.18 µm.

### Animal model

Forty two male Wistar rats (6 weeks old, 200±51 g) were randomly divided into three groups: control group (n = 6), LPS group (n = 18) and LPS+PFC group (n = 18). Rats in the LPS group were anesthetized by intraperitoneal injection with 2% pentobarbital at a dose of 40 mg/kg. The tracheas were gradually freed from surrounding tissues and instilled with 2 mg/mL of LPS (from *E. coli* O111:B4, Sigma, St. Louis, MO) at a dose of 1 mg/kg. The rats were placed upright and their bodies were rotated by hand to ensure good distribution of LPS in lung. Rats in LPS+PFC group received PFC emulsion through the femoral vein at 6 mL/kg, 30 min prior to LPS instillation as described above. At 2, 4 and 6 hours of exposure to LPS, 6 rats in the LPS group and 6 rats in LPS+PFC group were anesthetized by pentobarbital again and then blood was collected from abdominal aorta. After blood collection, the rats were sarcrificed right away and lung tissues were collected. In control group, the rats were treated the same as in LPS group, except LPS is substituted with an equal volume of normal saline (NS) and the rats were sacrificed 6 hours later.

### Blood gas analysis

One mL Blood from abdominal aorta was collected after 2, 4 and 6 hours of LPS treatment or 6 hours of NS treatment. PaO2 was measured by using a Radiometer ABL 625 Blood Gas Analyzer (Copenhagen, Denmark).

### Analysis of wet-to-dry weight ratio of lung tissue

After NS or LPS treatment, the rats were killed and the left lung was isolated. After blotting off blood and other contaminants, the wet weight of lung tissue was measured. Then the lung was dried in a 70°C oven for 72 h and the dry weight was measured. The wet/dry weight ratio of lung was calculated.

### Determination of myeloperoxidase expression in lung tissue

After the rats were sacrificed, the right lower lobe of lung was isolated and snap-frozen. The expression of myeloperoxidase (MPO) in lung was detected using enzyme-linked immunosorbent assay (ELISA, Bluegene, China) according to the manufacturer's instructions. Briefly, the lung tissue were homogenized and centrifuged at 12,000 g for 15 min at 4°C. The supernatants were added into a microtiter plate (100 µl/well) precoated with a murine anti-MPO mAb, then added 10 µl balance buffer and 50 µl enzyme conjugate to each well. After incubation for 1 h at 37°C, the plate was washed for 5 times followed by addition of the substrate and stop solution. Optical density (OD) at 450 nm was measured using a microplate reader. All the samples were assayed in triplicate.

### Detection of CD11b expression on polymorphonuclear neutrophils by flow Cytometry

Fifty µL of anticoagulated blood from abdominal aorta of rats was incubated with 10 µL of mouse anti-rat monoclonal CD11b-FITC antibody or mouse IgG_1_-FITC (Santa Cruz Biotechnology, Santa Cruz, USA) for 15 min in the dark at room temperature. 450 µL hemolysin was added to the mixture, followed by 10 min incubation. After centrifugation at 1200 rpm for 5 min, supernatant was discarded and the pellet was washed with phosphate buffered solution (PBS) buffer once and suspended in 450 µL of PBS buffer for flow Cytometry analysis. Data were analyzed using Cell Quest software (BD Biosciences). CD11b expression levels were presented as mean fluorescent intensity (MFI) of positive cells.

### Histology and ICAM-1 immunostaining

The upper and middle lobes of right lung were fixed in 10% formalin for 24 h. The tissues were dehydrated, embedded in paraffin and cut into 5 mm sections. The tissues were stained with hematoxylin and eosin (HE staining) after deparaffinization, and evaluated under an optical microscopy (Olympus BX51, Japan).

The expression of ICAM-1 in lung was determined by immunostaining. After deparaffinization and rehydrating, paraffin sections were placed into a pressure cooker containing antigen retrieval buffer (0.01 M citrate buffer, pH 6.0), cooked with full pressure for 2 minutes to unmask antigens. Immunostaining was performed by incubating the sections with mouse anti-rat ICAM-1 monoclonal antibody (1∶200, Abcam, MA) overnight at 4°C, biotin-conjugated secondary antibody (ZSGB-bio, China) at 37°C for 1 h, and streptavidin-HRP (ZSGB-bio, China) at 37°C for 30 min. 3,3-Diaminobenzidine (DAB, ZSGB-bio, China) was then used to visualize immunohistochemical staining. Cell nuclei were counterstained with hematoxylin. Images were obtained with an Olympus BX51 microscope and the proportion of positive staining cells was analyzed with Image-Pro plus 5.1 software. The expression of ICAM-1 in lung tissue was presented as mean photodensity.

For histology and immunostaining analysis, the slides were renamed by arabic number followed by a double-blinded examination by two pathologists.

### Statistical analysis

Data were analyzed using Statistical Product and Service Solutions (SPSS) statistical software version 13.0 (SPSS Inc., Chicago, IL) and expressed as mean ± standard deviations (SD). Within-group comparisons were analyzed using single factor analysis of variance and between–group comparisons were analyzed using one-sample t-test. Within group comparisons of results at different time intervals were analyzed using F test. *P* values of less than 0.05 were considered to be statistically significant.

## Results

### General observations

After 6 hours of treatment with normal saline or LPS, rats in the control group developed tachypnea that disappeared rapidly without any abnormal secretion, whereas the rats in LPS group exhibited tachypnea, cyanotic limbs and lips, mouth and nasal hemorrhage. The rats treated with LPS also had matted hair, slow reactions, listlessness and they rejected water. In addition, three of the rats in LPS group developed diarrhea. Rats in LPS+PFC group were more alert than those in LPS group, and they were able to escape capture as well as had no evidence of oral or nasal hemorrhage.

### Histopathological observation of lung tissue

**Figure 1:**
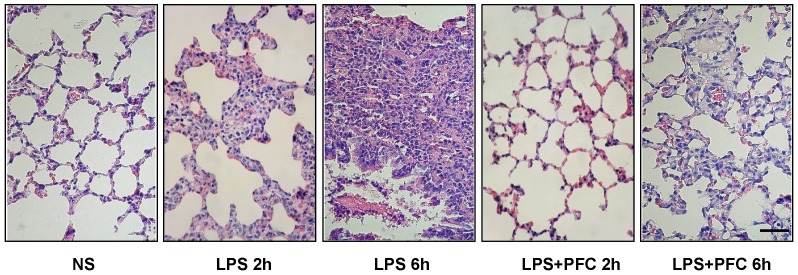
Histological examination of the lung sections from the rats treated by NS, LPS or LPS+PFC. The rats were treated with NS (n = 6) or LPS (n = 36) by intratracheal instillation. 18 rats received PFC through femoral vein prior to LPS instillation (LPS+PFC). Lung tissues were collected at the indicated time points after exposure to LPS and stained with HE. Scale bar, 25 µm.

We examined the histology of lung tissues of all the rats in this study by hematoxylin and eosin staining (Figure 1). There was no obvious difference in the lung tissue of rats treated with normal saline (NS) compared with normal untreated rats (data not shown). However, in LPS group the rats developed expansion and congestion of pulmonary minute vessels and alveolar septum capillaries. The alveolar walls burst, and the alveolar space was narrowed. In addition, partial alveoli were damaged and the alveolar septum was broadened. There was significant infiltration of PMN cells in the alveoli and pulmonary interstitial tissue, accompanied by hemorrhage and pulmonary interstitial edema. The injury got more and more serious with increased time of exposure to LPS. Importantly, we found that pretreatment of PFC emulsion markedly reduced the severity of pulmonary histopathological injury induced by LPS. In the lung of rats in LPS+PFC groups, there is wider alveolar space, less PMN cells infiltration, thinner alveolar septae and reduced hemorrhage comparison with the lung of rats only treated with LPS, as shown in Figure 1.

### Arterial blood gas analysis

**Figure 2:**
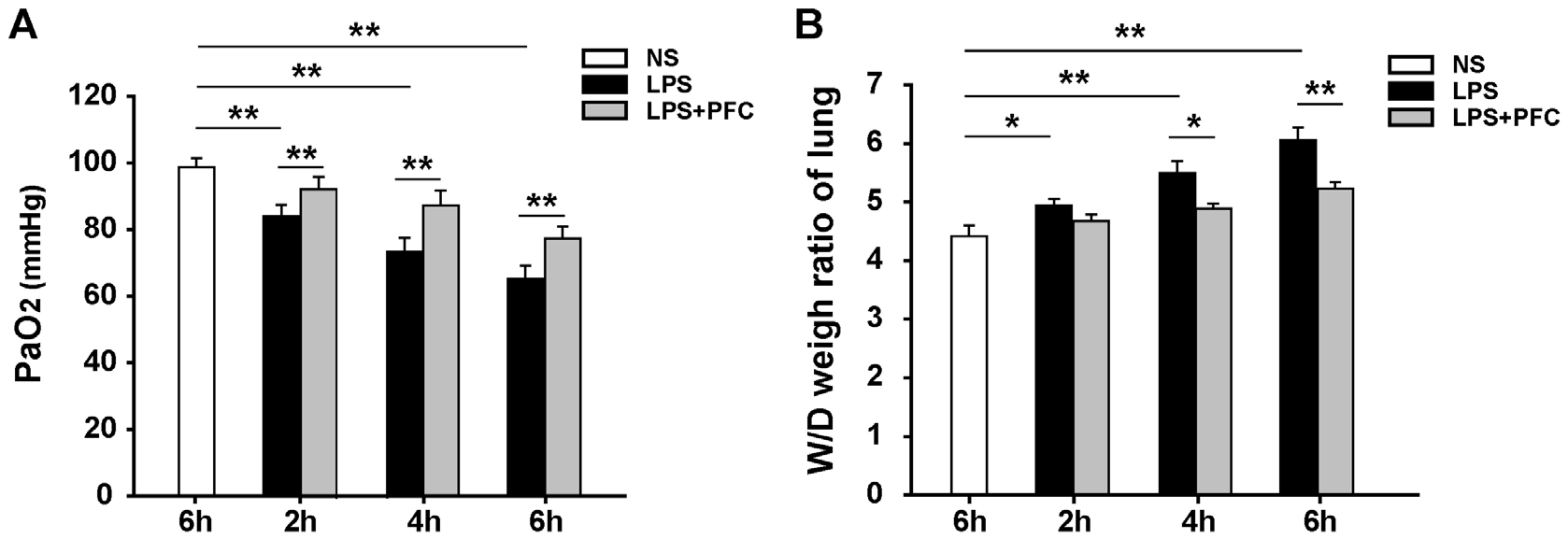
Pretreatment of PFC improves PaO_2_ and wet/dry weight ratio of lung in LPS induced acute lung injury. After the indicated time of NS or LPS exposure, PaO_2_ in the blood from abdominal aorta were measured (A); the wet/dry weight ratio of left lung in each rat was evaluated (B). Results are given as mean ± SD (n = 6). * *P*<0.05, ** *P*<0.01 between the indicated groups.

PaO2 is a measurement of the partial pressure of oxygen dissolved in plasma, which indicates how much oxygen was available in alveoli to dissolve in blood. We collected blood from abdominal aorta after NS or LPS treatment and analyzed PaO2. We found that PaO2 in the LPS group was significantly reduced compared to that in the control group (*P*<0.01) and it further decreased with longer exposure to LPS (Figure 2A). However, PFC treatment significantly increased PaO2 values in LPS+PFC group compared with LPS group after 2, 4 and 6 h of exposure to LPS (*P*<0.01) (Figure 2A), which may be due to improved O2 transport by PFC as well as the reduced lung injury as described above.

### Determination of lung wet-to-dry weight ratio

The lung wet-to-dry (W/D) weight ratio was used as an index of lung water accumulation, which is an indicator of the degree of lung edema. As shown in Fig 2B, the W/D ratio was significantly higher in LPS group compared with control group, and it further increased with longer time of exposure to LPS (*P*<0.05 or 0.01). After 4 and 6 hours of exposure to LPS, the lung W/D ratio in LPS+PFC group was significantly (*P*<0.05 or 0.01) lower than that in LPS group (Figure 2B), indicating that pretreatment of PFC could decrease the degress of lung edema induced by LPS. Together with lung histology and arterial blood gas analysis, these data for the first time demonstrated the therapeutic effect of intravenous infusion of PFC emulsion on LPS-induced acute lung injury in rats.

### Analysis of myeloperoxidase expression in lung tissue

**Figure 3:**
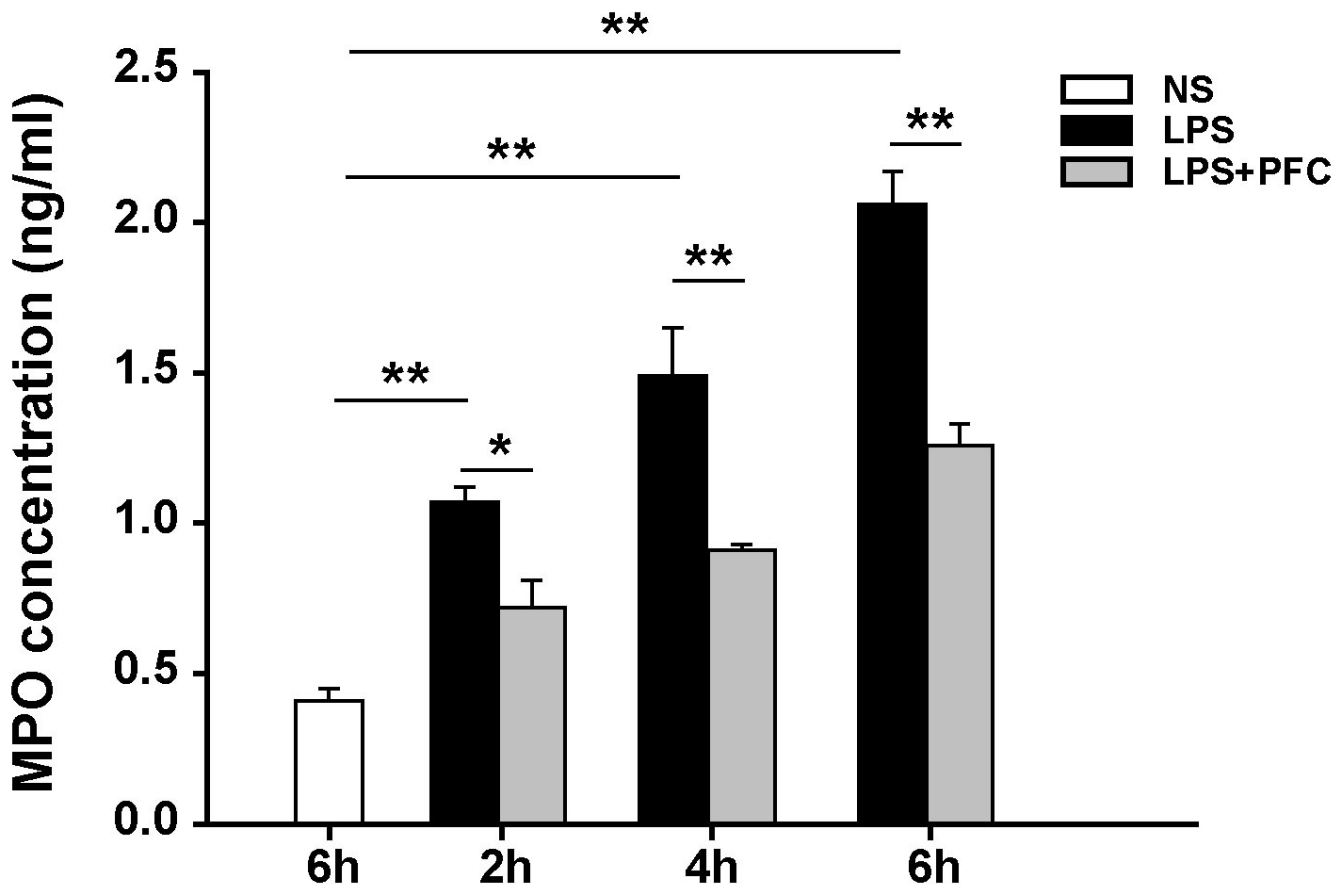
PFC reduces the expression of MPO in lung treated with LPS. After the indicated time of NS or LPS exposure, the right lower lobe of lung was isolated and the expression of myeloperoxidase (MPO) in lung was detected using enzyme-linked immunosorbent assay. Results are given as mean ± SD (n = 6). * *P*<0.05, ** *P*<0.01 between the indicated groups.

The expression of myeloperoxidase (MPO), which is an enzyme mainly presenting in azurophil granules of PMNs, was used as an index of the number of PMNs in the lung. It also reflected the degree of pulmonary retention and accumulation of PMN [30]. By using ELISA, we showed that the expression level of MPO in the lung was increased with LPS exposure time and its expression in LPS group was significantly (*P*<0.01) higher than that in the control group at all three exposure time points (Figure 3). However, the expression of MPO in the PFC group was significantly (*P*<0.05 or 0.01) lower than that in the LPS group. This result suggests that PFC treatment reduces infiltration of PMNs into lung tissue, which may explain the reduced inflammation and lung injury observed previously.

### Analysis of CD11b expression on polymorphonuclear neutrophils in circulation

The first step of recruitment of PMNs to the inflamed tissue is the attachment of circulating PMNs to vascular endothelial cells, which is mediated by the interaction between integrins expressed on PMNs, such as αMβ2, and cell adhesion molecules expressed on endothelial cells, such as intercellular adhesion molecule-1 (ICAM-1) [31]. CD11b is one protein subunit forming the heterodimeric integrin αMβ2 molecule, which mediates inflammation by regulating leukocyte adhesion and migration and has been implicated in several immune processes such as phagocytosis, cell-mediated cytotoxicity, chemotaxis and cellular activation [32].

**Figure 4:**
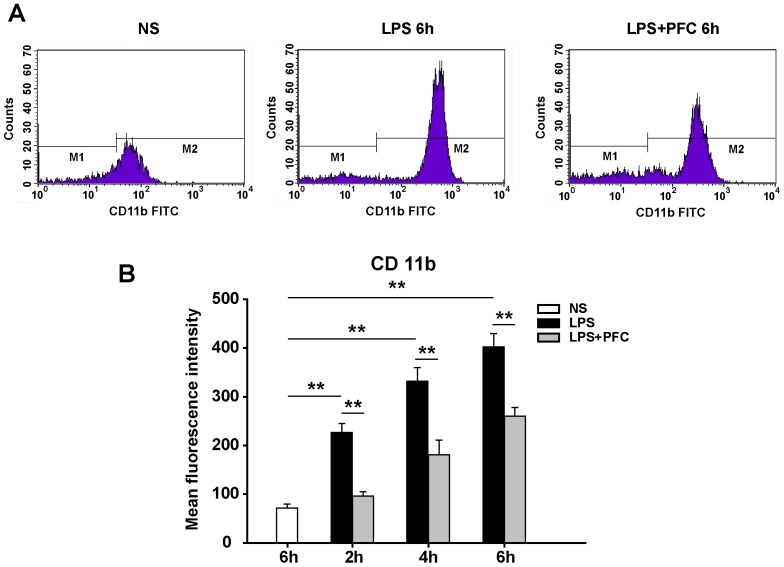
PFC reduces the expression of CD11b on circulating PMNs from rats treated with LPS. (A) Representative flow cytometry plots detecting CD11b on PMN cells at 6 h after NS or LPS exposure are shown. (B) Mean fluorescence intensity was measured to evaluate the expression of CD11b on PMN cells in different groups. Results are given as mean ± SD (n = 6). * *P*<0.05, ** *P*<0.01 between the indicated groups.

To reveal of the molecular mechanism of PFC's anti-inflammation effect, we analyzed the expression of CD11b on PMNs in abdominal aorta blood by flow Cytometry (Figure 4). We showed that the expression of CD11b was increased with the longer exposure to LPS. At 6 hours of exposure to LPS, the expression of CD11b in LPS group was 6-fold (*P*<0.01) higher compared with control group (Figure 4B). Interestingly, intravenous infusion of PFC emulsion significantly (*P*<0.01) decreased the expression of CD11b on circulating PMNs, in comparison with LPS group, at all three time points (Figure 4B).

### Analysis of ICAM-1 expression in lung tissue

**Figure 5:**
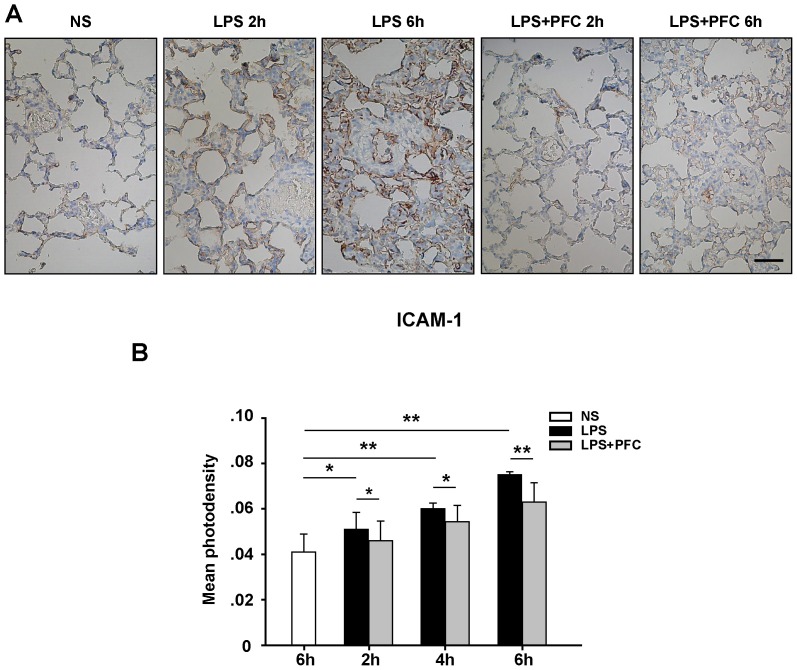
PFC attenuates the expression of ICAM-1 induced by LPS in lung tissue. (A) Representative immunohistochemistrical staining of ICAM-1 in lung tissue after NS or LPS exposure. Scale bar, 25 µm. (B) Mean photodenisity was measured to evaluate the expression of ICAM-1 in lung tissue. Results are given as mean ± SD (n = 6). * *P*<0.05, ** *P*<0.01 between the indicated groups.

Next, we characterized ICAM-1 expression in lung tissue by immunostaining and brown granules indicated ICAM-1 expression (Figure 5). In control group, low expression of ICAM-1 was observed on the surface of vascular endothelia, bronchiolar and alveolar epithelium (Figure 5A). In LPS group, claybank or dark brown granules were presented in the vascular, bronchiolar and alveolar epithelium after 2 h exposure to LPS, and this ICAM-1 signal markedly increased at 6 h of exposure to LPS (Figure 5A). However, in the rats treated with PFC, the lung exhibited MUCH weaker staining of ICAM-1 compared with the rats in LPS group (Figure 5A). Furthermore, we quantified these immunostaining with Image-Pro software and presented the expression of ICAM-1 in lung with mean photo densities, which were significantly (*P*<0.05 or 0.01) higher in LPS group than that in the control group (Figure 5B). The expression of ICAM-1 progressively increased from 2 hours to 6 hours of exposure to LPS (Figure 5B). PFC significantly inhibited the increase of ICAM-1 during the process of ALI and the mean photodensities of ICAM-1 in PFC group were markedly lower than that in LPS group (*P*<0.05 or 0.01) (Figure 5B).

Together, our data indicate that intravenous infusion of PFC emulsion reduces PMNs infiltrating into lung tissue by decreasing the expression of CD11b on circulating PMNs and ICAM-1 on vascular endothelia, bronchiolar and alveolar epithelium, which may explain its therapeutic effect on LPS induced ALI.

## Discussion

In this study, we for the first time demonstrated that intravenous infusion of PFC emulsion can alleviate lung injury using a LPS-induced ALI rat model. ALI/ARDS is a common, clinically intensive condition affecting the respiratory system. It is associated with high mortality and there is no effective treatment available to date [33]. Recently, PFC has been used as a novel way to prevent and treat ALI in the form of liquid ventilation (LV), partial liquid ventilation (PLV) or vaporized perfluorocarbon inhalation [23]–[25], [34], which can improve respiratory function and alleviates pulmonary injury and there was no significant adverse effect of PFC on lung, liver or kidney was observed in these studies [35], [36]. However, these interventions need establishment of artificial airway to facilitate PFC into the lungs, which is not suitable for treating patients with early ALI. Thus, in this study, we chose intravenous infusion of PFC emulsion and evaluated its therapeutic effect on ALI.

Here we showed that in a LPS-induced ALI rat model, intravenous infusion of PFC emulsion i) significantly improved lung function, indicated by increased arterial blood PaO_2_ and decreased lung wet-dry weight ratio (Figure 2); ii) reduced pulmonary injury, indicated by preserved lung structure (Figure 1) and decreased neutrophilic granulocyte infiltration into alveoli and pulmonary interstitial (Figure 3). These data indicate that intravenous infusion of PFC emulsion may be used to treat ALI, especially its early stage, in clinic.

There is good agreement that ALI/ARDS is an inflammatory injury of lung that mainly characterized by PMN infiltration. Enzymes, oxygen radicals and inflammatory mediators released from inflammatory cells were found to be the main factors associated with ALI [37]. The same study demonstrated that during ALI PMN cells adhered to vascular endothelium, crossed endothelial barrier and migrated to the region of inflammation. Based on these findings, it has been proposed that the interaction between inflammatory cells and adhesion molecules on the surface of vascular endothelia may be an important molecular mechanism responsible for ALI [38].

The mechanism of PFC protecting against ALI/ARDS remains controversial. It has been previously shown that PFC prevents PMN activation and decreases the synthesis and release of inflammatory mediators by macrophages [39], [40]. It may also serve as a physical barrier blocking the entrance of inflammatory cells and factors from alveoli into pulmonary parenchyma or coating the activated inflammatory cells and inhibiting their interaction with other activated cells [41]. Here we showed that PFC decreased the infiltration of PMN in lung by down-regulating the expression of CD11b on circulating PMNs (Figure 4) and ICAM-1 on endothelial cells (Figure 5), which is the core pathogenesis of ALI. In previous studies, both ICAM-1 and CD11b/CD18 have been shown to play a crucial role in the conjugation of PMN cells to the endothelium [42], [43]. CD11b and CD18 expressed by PMNs adhere to ICAM-1 on the surface of activated endothelia with high affinity [44]. This interaction mediates the migration and effusion of PMN cells, resulting in their accumulation at inflammatory region, which in turn strengthens and exaggerates inflammatory reaction, finally causing lung injury [45]. Wyman *et al.* found that ICAM-1 was not only needed to promote the pulmonary accumulation of PMNs, but also trigger the release of elastinase and other hydrolases from PMNs [46]. In line with these findings, it has been reported that ALI can be alleviated using ICAM-1 antibodies or genetic deletion of ICAM-1 in mice model [47].

Together, our study demonstrates the therapeutic effect of intravenous infusion of PFC emulsion on ALI. Importantly, this strategy avoids the establishment of artificial airway to facilitate PFC into the lungs as used in previous studies, thus it is more suitable for treating patients with early ALI. Moreover, we explored the molecular mechanism which may be responsible for this therapeutic effect of PFC. We showed that PFC decreases the infiltration of PMNs in lung by down-regulating the expression of CD11b on circulating PMNs and ICAM-1 on endothelial cells, which provides a theoretical foundation for using intravenous infusion of PFC to prevent and treat ALI/ARDS in clinical practice.

## References

[1] Ashbaugh DG, Bigelow DB, Petty TL, Levine BE (1967) Acute respiratory distress in adults. Lancet 2: 319–323. 4143721 10.1016/s0140-6736(67)90168-7

[2] Zhou X, Dai Q, Huang X (2012) Neutrophils in acute lung injury. Front Biosci 17: 2278–2283. 10.2741/4051 22652778

[3] Tsushima K, King LS, Aggarwal NR, Gorordo AD, D'Alessio FR, et al (2009) Acute Lung Injury Review. Intern Med 48: 621–630. 19420806 10.2169/internalmedicine.48.1741

[4] Lu Y, Song Z, Zhou X, Huang S, Zhu D, et al (2004) A 12-month clinical survey of incidence and outcome of acute respiratory distress syndrome in Shanghai intensive care units. Intensive Care Med 30: 2197–2203. 15650866 10.1007/s00134-004-2479-y

[5] Garber BG, H'ebert PC, Yelle JD, Hodder RV, McGowan J (1996) Adult respiratory distress syndrome: a systematic overview of incidence and risk factors. Crit Care Med 24: 687–695. 8612424 10.1097/00003246-199604000-00023

[6] Brower RG, Lanken PN, MacIntyre N, Matthay MA, Morris A, et al (2004) Higher versus lower positive end-expiratory pressures in patients with the acute respiratory distress syndrome. N Engl J Med 351: 327–336. 15269312 10.1056/NEJMoa032193

[7] Bollen CW, van Well GT, Sherry T, Beale RJ, Shah S, et al (2005) High frequency oscillatory ventilation compared with conventional mechanical ventilation in adult respiratory distress syndrome: a randomized controlled trial. Crit Care 9: R430–439. 16137357 10.1186/cc3737 PMC1269459

[8] Young D, Lamb SE, Shah S, MacKenzie I, Tunnicliffe W, et al (2013) High-frequency oscillation for acute respiratory distress syndrome. N Engl J Med 368: 806–813. 23339638 10.1056/NEJMoa1215716

[9] Hale DF, Cannon JW, Batchinsky AI, Cancio LC, Aden JK, et al (2012) Prone positioning improves oxygenation in adult burn patients with severe acute respiratory distress syndrome. J Trauma Acute Care Surg 72: 1634–1639. 22695433 10.1097/TA.0b013e318247cd4f

[10] Dickinson S, Park PK, Napolitano LM (2011) Prone-positioning therapy in ARDS. Crit Care Clin 27: 511–523. 21742215 10.1016/j.ccc.2011.05.010

[11] Taylor RW, Zimmerman JL, Dellinger RP, Straube RC, Criner GJ, et al (2004) Low-dose inhaled nitric oxide in patients with acute lung injury: a randomized controlled trial. JAMA 291: 1603–1609. 15069048 10.1001/jama.291.13.1603

[12] Angus DC, Clermont G, Linde-Zwirble WT, Musthafa AA, Dremsizov TT, et al (2006) Healthcare costs and long-term outcomes after acute respiratory distress syndrome: A phase III trial of inhaled nitric oxide. Crit Care Med 34: 2883–2890. 17075373 10.1097/01.CCM.0000248727.29055.25

[13] Thompson BT (2003) Glucocorticoids and acute lung injury. Crit Care Med 31: S253–257. 12682449 10.1097/01.CCM.0000057900.19201.55

[14] Perkins GD, McAuley DF, Thickett DR, Gao F (2006) The beta agonist lung injury trial (BALTI): a randomised placebo-controlled clinical trial. Am J Respir Crit Care Med 173: 281–287. 16254268 10.1164/rccm.200508-1302OC

[15] Gao Smith F, Perkins GD, Gates S, Young D, McAuley DF, et al (2012) BALTI-2 study investigators: Effect of intravenous β-2 agonist treatment on clinical outcomes in acute respiratory distress syndrome (BALTI-2): a multicentre, randomised controlled trial. Lancet 379: 229–235. 22166903 10.1016/S0140-6736(11)61623-1 PMC3266479

[16] Taut FJ, Rippin G, Schenk P, Findlay G, Wurst W, et al (2008) A Search for subgroups of patients with ARDS who may benefit from surfactant replacement therapy: a pooled analysis of five studies with recombinant surfactant protein-C surfactant (Venticute). Chest 134: 724–732. 18689599 10.1378/chest.08-0362

[17] Bernard G, Wheeler A, Naum C, Morris P, Nelson L, et al. (1999) A placebo controlled, randomised trail of IL-10 in acute lung injury (ALI). Chest 116 (Suppl): : 260S.

[18] Faithfull NS, Weers JG (1998) Perfluorocarbon compounds. Vox Sang 74 Suppl 2243–248. 9704451 10.1111/j.1423-0410.1998.tb05426.x

[19] Castro CI, Briceno JC (2010) Perfluorocarbon-based oxygen carriers: review of products and trials. Artif Organs 34: 622–634. 20698841 10.1111/j.1525-1594.2009.00944.x

[20] Greenspan JS, Wolfson MR, Shaffer TH (2000) Liquid ventilation. Semin Perinatol 24: 396–405. 11153901 10.1053/sper.2000.20092

[21] Riess JG (2006) Perfluorocarbon-based oxygen delivery. Artif Cells Blood Substit Immobil Biotechnol 34: 567–580. 17090429 10.1080/10731190600973824

[22] Greenspan JS, Wolfson MR, Rubenstein SD, Shaffer TH (1990) Liquid ventilation of human preterm neonates. J Pediatr 117: 106–111. 2115078 10.1016/s0022-3476(05)82457-6

[23] Hadjiliadis D (2000) Partial liquid ventilation. Semin Respir Crit Care Med 21: 175–181. 16088730 10.1055/s-2000-9852

[24] Yoxall CW, Subhedar NV, Shaw NJ (1997) Liquid ventilation in the preterm neonate. Thorax 52 Suppl 3S3–8. 9381423 10.1136/thx.52.2008.s3 PMC1765887

[25] Wang X, Zhang J, Li X, Liu Y, Yang H, et al. (2013) Sustained Improvement of Gas Exchange and Lung Mechanics by Vaporized Perfluorocarbon Inhalation in Piglet Acute Lung Injury Model. Clin Respir J. 2013 Sep 13. doi:10.1111/crj.12053. [Epub ahead of print] 10.1111/crj.12053 24028088

[26] Spahn DR (1999) Blood substitutes. Artificial oxygen carriers: perfluorocarbon emulsions. Crit Care 3: R93–97. 11094488 10.1186/cc364 PMC137239

[27] Kerins DM (1994) Role of perfluorocarbon Fluosol-DA in coronary angioplasty. Am J Med Sci 307: 218–221. 8160713 10.1097/00000441-199403000-00009

[28] Spiess BD (2009) Perfluorocarbon emulsions as a promising technology: a review of tissue and vascular gas dynamics. J Appl Physiol 106: 1444–52. 19179651 10.1152/japplphysiol.90995.2008

[29] Keipert PE (2001) Perflubron emulsion (Oxygent(tm)): a temporary intravenous oxygen carrier. Anasthesiol Intensivmed Notfallmed Schmerzther 36 Suppl 2S104–106. 11753711 10.1055/s-2001-18189

[30] Sharma J, Rastogi P, Creer MH, McHowat J (2009) Polymorphonuclear leukocytes isolated from umbilical cord blood as a useful research tool to study adherence to cell monolayers. J Immunol Methods 351: 30–35. 19800347 10.1016/j.jim.2009.09.008 PMC2783264

[31] Butcher EC (1991) Leukocyte-endothelial cell recognition: three (or more) steps to specificity and diversity. Cell 67: 1033–1036. 1760836 10.1016/0092-8674(91)90279-8

[32] Solovjov DA, Pluskota E, Plow EF (2005) Distinct roles for the alpha and beta subunits in the functions of integrin alphaMbeta2. J Biol Chem 280: 1336–45. 15485828 10.1074/jbc.M406968200

[33] Shen Y, Wang D, Wang X (2011) Role of CCR2 and IL-8 in acute lung injury: a new mechanism and therapeutic target. Expert Rev Respir Med 5: 107–114. 21348591 10.1586/ers.10.80

[34] Wolfson MR, Shaffer TH (2004) Liquid ventilation: an adjunct for respiratory management. Paediatr Anaesth 14: 15–23. 14717869 10.1046/j.1460-9592.2003.01206.x

[35] Albaghdadi AS, Brooks LA, Pretorius AM, Kerber RE (2010) Perfluorocarbon induced intra-arrest hypothermia does not improve survival in a swine model of asphyxial cardiac arrest. Resuscitation 81: 353–358. 20044200 10.1016/j.resuscitation.2009.11.018 PMC2827481

[36] Shashikant BN, Miller TL, Jeng MJ, Davis J, Shaffer TH, et al (2005) Differential impact of perfluorochemical physical properties on the physiologic, histologic, and inflammatory profile in acute lung injury. Crit Care Med 33: 1096–1103. 15891342 10.1097/01.ccm.0000163218.79770.29

[37] Everhart MB, Han W, Sherrill TP, Arutiunov M, Polosukhin VV, et al (2006) Duration and intensity of NF-kappaB activity determine the severity of endotoxin-induced acute lung injury. J Immunol 176: 4995–5005. 16585596 10.4049/jimmunol.176.8.4995

[38] Chopra M, Reuben JS, Sharma AC (2009) Acute lung injury:apoptosis and signaling mechanisms. Exp Biol Med 234: 361–371. 10.3181/0811-MR-318 19176873

[39] Gale SC, Gorman GD, Copeland JG, McDonagh PF (2007) Perflubron emulsion prevents PMN activation and improves myocardial functional recovery after cold ischemia and reperfusion. J Surg Res 138: 135–140. 17173933 10.1016/j.jss.2006.08.029

[40] Chang H, Kuo FC, Lai YS, Chou TC (2005) Inhibition of inflammatory responses by FC-77, a perfluorochemical, in lipopolysaccharide-treated RAW 264.7 macrophages. Intensive Care Med 31: 977–984. 15931525 10.1007/s00134-005-2652-y

[41] Baba A, Kim YK, Zhang H, Liu M, Slutsky AS (2000) Perfluorocarbon blocks tumor necrosis factor-alpha-induced interleukin-8 release from alveolar epithelial cells in vitro. Crit Care Med 28: 1113–1118. 10809292 10.1097/00003246-200004000-00034

[42] Michetti C, Coimbra R, Hoyt DB, Loomis W, Junger W, et al (2003) Pentoxifylline reduces acute lung injury in chronic endotoxemia. J Surg Res 115: 92–99. 14572778 10.1016/s0022-4804(03)00219-1

[43] Zhang X, Wu D, Jiang X (2009) Icam-1 and acute pancreatitis complicated by acute lung injury. JOP 10: 8–14. 19129609

[44] Gardinali M, Borrelli E, Chiara O, Lundberg C, Padalino P, et al (2000) Inhibition of CD11-CD18 complex prevents acute lung injury and reduces mortality after peritonitis in rabbits. Am J Respir Crit Care Med 161: 1022–1029. 10712358 10.1164/ajrccm.161.3.9901066

[45] Ma XG, Cao XY (2006) Protective effects of colquhounia root tablet against oleic acid induced acute lung injury in rats. Chin J Anesthesiol 26: 363–365.

[46] Wyman TH, Bjornsen AJ, Elzi DJ, Smith CW, England KM, et al (2002) A two-insult in vitro model of PMN-mediated pulmonary endothelial damage: requirements for adherence and chemokine release. Am J Physiol Cell Physiol 283: C1592–1603. 12388071 10.1152/ajpcell.00540.2001

[47] Doerschuk CM, Quinlan WM, Doyle NA, Bullard DC, Vestweber D, et al (1996) The role of P-selectin and ICAM-1 in acute lung injury as determined using blocking antibodies and mutant mice. J Immunol 157: 4609–4614. 8906840

